# Supplementary material for: OpenMM 7: Rapid development of high performance algorithms for molecular dynamics
Source: PLoS Comput Biol. 2017 Jul 26;13(7):e1005659. doi: 10.1371/journal.pcbi.1005659 (PMC5549999; doi:10.1371/journal.pcbi.1005659)
Supplement: S1 Source Code — (ZIP) [file pcbi.1005659.s002.zip › OpenMM-7.0.1-Source/docs/api-c++/generated/OpenMM.CustomHbondForce.html]

CustomHbondForce — OpenMM @OPENMM\_MAJOR\_VERSION@.@OPENMM\_MINOR\_VERSION@ documentation


# CustomHbondForce¶

*class* `OpenMM::``CustomHbondForce`¶
:   This class supports a wide variety of energy functions used to represent hydrogen bonding. It computes interactions between “donor” particle groups and “acceptor” particle groups, where each group may include up to three particles. Typically a donor group consists of a hydrogen atom and the atoms it is bonded to, and an acceptor group consists of a negatively charged atom and the atoms it is bonded to.

    We refer to the particles in a donor group as d1, d2 and d3, and the particles in an acceptor group as a1, a2, and a3. For each donor and each acceptor, `CustomHbondForce` evaluates a user supplied algebraic expression to determine the interaction energy. The expression may depend on arbitrary distances, angles, and dihedral angles defined by any of the six particles involved. The function distance(p1, p2) is the distance between the particles p1 and p2 (where “p1” and “p2” should be replaced by the names of the actual particles to calculate the distance between), angle(p1, p2, p3) is the angle formed by the three specified particles, and dihedral(p1, p2, p3, p4) is the dihedral angle formed by the four specified particles.

    The expression also may involve tabulated functions, and may depend on arbitrary global, per-donor, and per-acceptor parameters. It also optionally supports periodic boundary conditions and cutoffs for long range interactions.

    To use this class, create a `CustomHbondForce` object, passing an algebraic expression to the constructor that defines the interaction energy between each donor and acceptor. Then call `addPerDonorParameter()` to define per-donor parameters, `addPerAcceptorParameter()` to define per-acceptor parameters, and `addGlobalParameter()` to define global parameters. The values of per-donor and per-acceptor parameters are specified as part of the system definition, while values of global parameters may be modified during a simulation by calling `Context::setParameter()`.

    Next, call `addDonor()` and `addAcceptor()` to define donors and acceptors and specify their parameter values. After a donor or acceptor has been added, you can modify its parameters by calling `setDonorParameters()` or `setAcceptorParameters()`. This will have no effect on Contexts that already exist unless you call `updateParametersInContext()`.
    `CustomHbondForce` also lets you specify “exclusions”, particular combinations of donors and acceptors whose interactions should be omitted from force and energy calculations. This is most often used for particles that are bonded to each other.

    As an example, the following code creates a `CustomHbondForce` that implements a simple harmonic potential to keep the distance between a1 and d1, and the angle formed by a1-d1-d2, near ideal values:

    ```
    CustomHbondForce* force = new CustomHbondForce("k*(distance(a1,d1)-r0)^2*(angle(a1,d1,d2)-theta0)^2");
    ```

    This force depends on three parameters: k, r0, and theta0. The following code defines these as per-donor parameters:

    ```
    force->addPerDonorParameter("k");
    force->addPerDonorParameter("r0");
    force->addPerDonorParameter("theta0");
    ```

    Expressions may involve the operators + (add), - (subtract), \* (multiply), / (divide), and ^ (power), and the following functions: sqrt, exp, log, sin, cos, sec, csc, tan, cot, asin, acos, atan, sinh, cosh, tanh, erf, erfc, min, max, abs, floor, ceil, step, delta, select. All trigonometric functions are defined in radians, and log is the natural logarithm. step(x) = 0 if x is less than 0, 1 otherwise. delta(x) = 1 if x is 0, 0 otherwise. select(x,y,z) = z if x = 0, y otherwise.

    In addition, you can call `addTabulatedFunction()` to define a new function based on tabulated values. You specify the function by creating a `TabulatedFunction` object. That function can then appear in the expression.

    Methods

    |  |  |
    | --- | --- |
    | `CustomHbondForce` | Create a `CustomHbondForce`. |
    | `~CustomHbondForce` |  |
    | `getNumDonors` | Get the number of donors for which force field parameters have been defined. |
    | `getNumAcceptors` | Get the number of acceptors for which force field parameters have been defined. |
    | `getNumExclusions` | Get the number of donor-acceptor pairs whose interactions should be excluded. |
    | `getNumPerDonorParameters` | Get the number of per-donor parameters that the interaction depends on. |
    | `getNumPerAcceptorParameters` | Get the number of per-acceptor parameters that the interaction depends on. |
    | `getNumGlobalParameters` | Get the number of global parameters that the interaction depends on. |
    | `getNumTabulatedFunctions` | Get the number of tabulated functions that have been defined. |
    | `getNumFunctions` | Get the number of tabulated functions that have been defined. |
    | `getEnergyFunction` | Get the algebraic expression that gives the interaction energy between a donor and an acceptor |
    | `setEnergyFunction` | Set the algebraic expression that gives the interaction energy between a donor and an acceptor |
    | `getNonbondedMethod` | Get the method used for handling long range nonbonded interactions. |
    | `setNonbondedMethod` | Set the method used for handling long range nonbonded interactions. |
    | `getCutoffDistance` | Get the cutoff distance (in nm) being used. |
    | `setCutoffDistance` | Set the cutoff distance (in nm) being used. |
    | `addPerDonorParameter` | Add a new per-donor parameter that the interaction may depend on. |
    | `getPerDonorParameterName` | Get the name of a per-donor parameter. |
    | `setPerDonorParameterName` | Set the name of a per-donor parameter. |
    | `addPerAcceptorParameter` | Add a new per-acceptor parameter that the interaction may depend on. |
    | `getPerAcceptorParameterName` | Get the name of a per-acceptor parameter. |
    | `setPerAcceptorParameterName` | Set the name of a per-acceptor parameter. |
    | `addGlobalParameter` | Add a new global parameter that the interaction may depend on. |
    | `getGlobalParameterName` | Get the name of a global parameter. |
    | `setGlobalParameterName` | Set the name of a global parameter. |
    | `getGlobalParameterDefaultValue` | Get the default value of a global parameter. |
    | `setGlobalParameterDefaultValue` | Set the default value of a global parameter. |
    | `addDonor` | Add a donor group to the force |
    | `getDonorParameters` | Get the properties of a donor group. |
    | `setDonorParameters` | Set the properties of a donor group. |
    | `addAcceptor` | Add an acceptor group to the force |
    | `getAcceptorParameters` | Get the properties of an acceptor group. |
    | `setAcceptorParameters` | Set the properties of an acceptor group. |
    | `addExclusion` | Add a donor-acceptor pair to the list of interactions that should be excluded. |
    | `getExclusionParticles` | Get the donor and acceptor in a pair whose interaction should be excluded. |
    | `setExclusionParticles` | Get the donor and acceptor in a pair whose interaction should be excluded. |
    | `addTabulatedFunction` | Add a tabulated function that may appear in the energy expression. |
    | `getTabulatedFunction` | Get a const reference to a tabulated function that may appear in the energy expression. |
    | `getTabulatedFunction` | Get a reference to a tabulated function that may appear in the energy expression. |
    | `getTabulatedFunctionName` | Get the name of a tabulated function that may appear in the energy expression. |
    | `addFunction` | Add a tabulated function that may appear in the energy expression. |
    | `getFunctionParameters` | Get the parameters for a tabulated function that may appear in the energy expression. |
    | `setFunctionParameters` | Set the parameters for a tabulated function that may appear in the energy expression. |
    | `updateParametersInContext` | Update the per-donor and per-acceptor parameters in a `Context` to match those stored in this `Force` object. |
    | `usesPeriodicBoundaryConditions` | Returns whether or not this force makes use of periodic boundary conditions. |

    Enum: NonbondedMethod

    |  |  |
    | --- | --- |
    | **NoCutoff** | No cutoff is applied to nonbonded interactions. The full set of N^2 interactions is computed exactly. This necessarily means that periodic boundary conditions cannot be used. This is the default. |
    | **CutoffNonPeriodic** | Interactions beyond the cutoff distance are ignored. |
    | **CutoffPeriodic** | Periodic boundary conditions are used, so that each particle interacts only with the nearest periodic copy of each other particle. Interactions beyond the cutoff distance are ignored. |

    `CustomHbondForce`(*const* std::string &*energy*)¶
    :   Create a `CustomHbondForce`.

        |  |  |
        | --- | --- |
        | Parameters: |  |

        - **energy** – an algebraic expression giving the interaction energy between a donor and an acceptor as a function of inter-particle distances, angles, and dihedrals, as well as any global, per-donor, and per-acceptor parameters

    `~CustomHbondForce`()¶

    int `getNumDonors`() *const*¶
    :   Get the number of donors for which force field parameters have been defined.

    int `getNumAcceptors`() *const*¶
    :   Get the number of acceptors for which force field parameters have been defined.

    int `getNumExclusions`() *const*¶
    :   Get the number of donor-acceptor pairs whose interactions should be excluded.

    int `getNumPerDonorParameters`() *const*¶
    :   Get the number of per-donor parameters that the interaction depends on.

    int `getNumPerAcceptorParameters`() *const*¶
    :   Get the number of per-acceptor parameters that the interaction depends on.

    int `getNumGlobalParameters`() *const*¶
    :   Get the number of global parameters that the interaction depends on.

    int `getNumTabulatedFunctions`() *const*¶
    :   Get the number of tabulated functions that have been defined.

    int `getNumFunctions`() *const*¶
    :   Get the number of tabulated functions that have been defined.

        Deprecated

        This method exists only for backward compatibility. Use `getNumTabulatedFunctions()` instead.

    *const* std::string &`getEnergyFunction`() *const*¶
    :   Get the algebraic expression that gives the interaction energy between a donor and an acceptor

    void `setEnergyFunction`(*const* std::string &*energy*)¶
    :   Set the algebraic expression that gives the interaction energy between a donor and an acceptor

    NonbondedMethod `getNonbondedMethod`() *const*¶
    :   Get the method used for handling long range nonbonded interactions.

    void `setNonbondedMethod`(NonbondedMethod *method*)¶
    :   Set the method used for handling long range nonbonded interactions.

    double `getCutoffDistance`() *const*¶
    :   Get the cutoff distance (in nm) being used. All interactions for which the distance between d1 and a1 is greater than the cutoff will be ignored. If the NonbondedMethod in use is NoCutoff, this value will have no effect.

        |  |  |
        | --- | --- |
        | Returns: | the cutoff distance, measured in nm |

    void `setCutoffDistance`(double *distance*)¶
    :   Set the cutoff distance (in nm) being used. All interactions for which the distance between d1 and a1 is greater than the cutoff will be ignored. If the NonbondedMethod in use is NoCutoff, this value will have no effect.

        |  |  |
        | --- | --- |
        | Parameters: |  |

        - **distance** – the cutoff distance, measured in nm

    int `addPerDonorParameter`(*const* std::string &*name*)¶
    :   Add a new per-donor parameter that the interaction may depend on.

        |  |  |
        | --- | --- |
        | Parameters: |  |

        - **name** – the name of the parameter

        |  |  |
        | --- | --- |
        | Returns: | the index of the parameter that was added |

    *const* std::string &`getPerDonorParameterName`(int *index*) *const*¶
    :   Get the name of a per-donor parameter.

        |  |  |
        | --- | --- |
        | Parameters: |  |

        - **index** – the index of the parameter for which to get the name

        |  |  |
        | --- | --- |
        | Returns: | the parameter name |

    void `setPerDonorParameterName`(int *index*, *const* std::string &*name*)¶
    :   Set the name of a per-donor parameter.

        |  |  |
        | --- | --- |
        | Parameters: |  |

        - **index** – the index of the parameter for which to set the name
        - **name** – the name of the parameter

    int `addPerAcceptorParameter`(*const* std::string &*name*)¶
    :   Add a new per-acceptor parameter that the interaction may depend on.

        |  |  |
        | --- | --- |
        | Parameters: |  |

        - **name** – the name of the parameter

        |  |  |
        | --- | --- |
        | Returns: | the index of the parameter that was added |

    *const* std::string &`getPerAcceptorParameterName`(int *index*) *const*¶
    :   Get the name of a per-acceptor parameter.

        |  |  |
        | --- | --- |
        | Parameters: |  |

        - **index** – the index of the parameter for which to get the name

        |  |  |
        | --- | --- |
        | Returns: | the parameter name |

    void `setPerAcceptorParameterName`(int *index*, *const* std::string &*name*)¶
    :   Set the name of a per-acceptor parameter.

        |  |  |
        | --- | --- |
        | Parameters: |  |

        - **index** – the index of the parameter for which to set the name
        - **name** – the name of the parameter

    int `addGlobalParameter`(*const* std::string &*name*, double *defaultValue*)¶
    :   Add a new global parameter that the interaction may depend on.

        |  |  |
        | --- | --- |
        | Parameters: |  |

        - **name** – the name of the parameter
        - **defaultValue** – the default value of the parameter

        |  |  |
        | --- | --- |
        | Returns: | the index of the parameter that was added |

    *const* std::string &`getGlobalParameterName`(int *index*) *const*¶
    :   Get the name of a global parameter.

        |  |  |
        | --- | --- |
        | Parameters: |  |

        - **index** – the index of the parameter for which to get the name

        |  |  |
        | --- | --- |
        | Returns: | the parameter name |

    void `setGlobalParameterName`(int *index*, *const* std::string &*name*)¶
    :   Set the name of a global parameter.

        |  |  |
        | --- | --- |
        | Parameters: |  |

        - **index** – the index of the parameter for which to set the name
        - **name** – the name of the parameter

    double `getGlobalParameterDefaultValue`(int *index*) *const*¶
    :   Get the default value of a global parameter.

        |  |  |
        | --- | --- |
        | Parameters: |  |

        - **index** – the index of the parameter for which to get the default value

        |  |  |
        | --- | --- |
        | Returns: | the parameter default value |

    void `setGlobalParameterDefaultValue`(int *index*, double *defaultValue*)¶
    :   Set the default value of a global parameter.

        |  |  |
        | --- | --- |
        | Parameters: |  |

        - **index** – the index of the parameter for which to set the default value
        - **defaultValue** – the default value of the parameter

    int `addDonor`(int *d1*, int *d2*, int *d3*, *const* std::vector<double> &*parameters* = std::vector< double >())¶
    :   Add a donor group to the force

        |  |  |
        | --- | --- |
        | Parameters: |  |

        - **d1** – the index of the first particle for this donor group
        - **d2** – the index of the second particle for this donor group. If the group only includes one particle, this must be -1.
        - **d3** – the index of the third particle for this donor group. If the group includes less than three particles, this must be -1.
        - **parameters** – the list of per-donor parameter values for the new donor

        |  |  |
        | --- | --- |
        | Returns: | the index of the donor that was added |

    void `getDonorParameters`(int *index*, int &*d1*, int &*d2*, int &*d3*, std::vector<double> &*parameters*) *const*¶
    :   Get the properties of a donor group.

        |  |  |
        | --- | --- |
        | Parameters: |  |

        - **index** – the index of the donor group to get
        - **d1** – [out] the index of the first particle for this donor group
        - **d2** – [out] the index of the second particle for this donor group. If the group only includes one particle, this will be -1.
        - **d3** – [out] the index of the third particle for this donor group. If the group includes less than three particles, this will be -1.
        - **parameters** – [out] the list of per-donor parameter values for the donor

    void `setDonorParameters`(int *index*, int *d1*, int *d2*, int *d3*, *const* std::vector<double> &*parameters* = std::vector< double >())¶
    :   Set the properties of a donor group.

        |  |  |
        | --- | --- |
        | Parameters: |  |

        - **index** – the index of the donor group to set
        - **d1** – the index of the first particle for this donor group
        - **d2** – the index of the second particle for this donor group. If the group only includes one particle, this must be -1.
        - **d3** – the index of the third particle for this donor group. If the group includes less than three particles, this must be -1.
        - **parameters** – the list of per-donor parameter values for the donor

    int `addAcceptor`(int *a1*, int *a2*, int *a3*, *const* std::vector<double> &*parameters* = std::vector< double >())¶
    :   Add an acceptor group to the force

        |  |  |
        | --- | --- |
        | Parameters: |  |

        - **a1** – the index of the first particle for this acceptor group
        - **a2** – the index of the second particle for this acceptor group. If the group only includes one particle, this must be -1.
        - **a3** – the index of the third particle for this acceptor group. If the group includes less than three particles, this must be -1.
        - **parameters** – the list of per-acceptor parameter values for the new acceptor

        |  |  |
        | --- | --- |
        | Returns: | the index of the acceptor that was added |

    void `getAcceptorParameters`(int *index*, int &*a1*, int &*a2*, int &*a3*, std::vector<double> &*parameters*) *const*¶
    :   Get the properties of an acceptor group.

        |  |  |
        | --- | --- |
        | Parameters: |  |

        - **index** – the index of the acceptor group to get
        - **a1** – [out] the index of the first particle for this acceptor group
        - **a2** – [out] the index of the second particle for this acceptor group. If the group only includes one particle, this will be -1.
        - **a3** – [out] the index of the third particle for this acceptor group. If the group includes less than three particles, this will be -1.
        - **parameters** – [out] the list of per-acceptor parameter values for the acceptor

    void `setAcceptorParameters`(int *index*, int *a1*, int *a2*, int *a3*, *const* std::vector<double> &*parameters* = std::vector< double >())¶
    :   Set the properties of an acceptor group.

        |  |  |
        | --- | --- |
        | Parameters: |  |

        - **index** – the index of the acceptor group to set
        - **a1** – the index of the first particle for this acceptor group
        - **a2** – the index of the second particle for this acceptor group. If the group only includes one particle, this must be -1.
        - **a3** – the index of the third particle for this acceptor group. If the group includes less than three particles, this must be -1.
        - **parameters** – the list of per-acceptor parameter values for the acceptor

    int `addExclusion`(int *donor*, int *acceptor*)¶
    :   Add a donor-acceptor pair to the list of interactions that should be excluded.

        |  |  |
        | --- | --- |
        | Parameters: |  |

        - **donor** – the index of the donor to exclude
        - **acceptor** – the index of the acceptor to exclude

        |  |  |
        | --- | --- |
        | Returns: | the index of the exclusion that was added |

    void `getExclusionParticles`(int *index*, int &*donor*, int &*acceptor*) *const*¶
    :   Get the donor and acceptor in a pair whose interaction should be excluded.

        |  |  |
        | --- | --- |
        | Parameters: |  |

        - **index** – the index of the exclusion for which to get donor and acceptor indices
        - **donor** – [out] the index of the donor
        - **acceptor** – [out] the index of the acceptor

    void `setExclusionParticles`(int *index*, int *donor*, int *acceptor*)¶
    :   Get the donor and acceptor in a pair whose interaction should be excluded.

        |  |  |
        | --- | --- |
        | Parameters: |  |

        - **index** – the index of the exclusion for which to get donor and acceptor indices
        - **donor** – the index of the donor
        - **acceptor** – the index of the acceptor

    int `addTabulatedFunction`(*const* std::string &*name*, TabulatedFunction \**function*)¶
    :   Add a tabulated function that may appear in the energy expression.

        |  |  |
        | --- | --- |
        | Parameters: |  |

        - **name** – the name of the function as it appears in expressions
        - **function** – a `TabulatedFunction` object defining the function. The `TabulatedFunction` should have been created on the heap with the “new” operator. The `Force` takes over ownership of it, and deletes it when the `Force` itself is deleted.

        |  |  |
        | --- | --- |
        | Returns: | the index of the function that was added |

    *const* TabulatedFunction &`getTabulatedFunction`(int *index*) *const*¶
    :   Get a const reference to a tabulated function that may appear in the energy expression.

        |  |  |
        | --- | --- |
        | Parameters: |  |

        - **index** – the index of the function to get

        |  |  |
        | --- | --- |
        | Returns: | the `TabulatedFunction` object defining the function |

    TabulatedFunction &`getTabulatedFunction`(int *index*)¶
    :   Get a reference to a tabulated function that may appear in the energy expression.

        |  |  |
        | --- | --- |
        | Parameters: |  |

        - **index** – the index of the function to get

        |  |  |
        | --- | --- |
        | Returns: | the `TabulatedFunction` object defining the function |

    *const* std::string &`getTabulatedFunctionName`(int *index*) *const*¶
    :   Get the name of a tabulated function that may appear in the energy expression.

        |  |  |
        | --- | --- |
        | Parameters: |  |

        - **index** – the index of the function to get

        |  |  |
        | --- | --- |
        | Returns: | the name of the function as it appears in expressions |

    int `addFunction`(*const* std::string &*name*, *const* std::vector<double> &*values*, double *min*, double *max*)¶
    :   Add a tabulated function that may appear in the energy expression.

        Deprecated

        This method exists only for backward compatibility. Use `addTabulatedFunction()` instead.

    void `getFunctionParameters`(int *index*, std::string &*name*, std::vector<double> &*values*, double &*min*, double &*max*) *const*¶
    :   Get the parameters for a tabulated function that may appear in the energy expression.

        Deprecated

        This method exists only for backward compatibility. Use getTabulatedFunctionParameters() instead. If the specified function is not a `Continuous1DFunction`, this throws an exception.

    void `setFunctionParameters`(int *index*, *const* std::string &*name*, *const* std::vector<double> &*values*, double *min*, double *max*)¶
    :   Set the parameters for a tabulated function that may appear in the energy expression.

        Deprecated

        This method exists only for backward compatibility. Use setTabulatedFunctionParameters() instead. If the specified function is not a `Continuous1DFunction`, this throws an exception.

    void `updateParametersInContext`(Context &*context*)¶
    :   Update the per-donor and per-acceptor parameters in a `Context` to match those stored in this `Force` object. This method provides an efficient method to update certain parameters in an existing `Context` without needing to reinitialize it. Simply call `setDonorParameters()` and `setAcceptorParameters()` to modify this object’s parameters, then call `updateParametersInContext()` to copy them over to the `Context`.

        This method has several limitations. The only information it updates is the values of per-donor and per-acceptor parameters. All other aspects of the `Force` (the energy function, nonbonded method, cutoff distance, etc.) are unaffected and can only be changed by reinitializing the `Context`. The set of particles involved in a donor or acceptor cannot be changed, nor can new donors or acceptors be added.

    bool `usesPeriodicBoundaryConditions`() *const*¶
    :   Returns whether or not this force makes use of periodic boundary conditions.

        |  |  |
        | --- | --- |
        | Returns: | true if force uses PBC and false otherwise |

High performance molecular simulation on GPUs


### Navigation

- Core Objects
- Forces
- Integrators
- Extras

©2015, Stanford University and the Authors.
|
Powered by Sphinx 1.3.1
& Alabaster 0.7.7
|
Page source
